# Pharmacokinetic parameters and radiomics model based on dynamic contrast enhanced MRI for the preoperative prediction of sentinel lymph node metastasis in breast cancer

**DOI:** 10.1186/s40644-020-00342-x

**Published:** 2020-09-15

**Authors:** Meijie Liu, Ning Mao, Heng Ma, Jianjun Dong, Kun Zhang, Kaili Che, Shaofeng Duan, Xuexi Zhang, Yinghong Shi, Haizhu Xie

**Affiliations:** 1 School of Clinical Medicine, Binzhou Medical University, Yantai, Shandong P. R. China 264000; 2 Department of Radiology, Yantai Yuhuangding Hospital, No. 20 Yuhuangding road, Yantai, Shandong P. R. China 264000; 3 GE Healthcare, China, Shanghai, P. R. China 200000

**Keywords:** Breast cancer, Sentinel lymph node, Magnetic resonance imaging, Radiomics, Pharmacokinetic parameters

## Abstract

**Background:**

To establish pharmacokinetic parameters and a radiomics model based on dynamic contrast enhanced magnetic resonance imaging (DCE-MRI) for predicting sentinel lymph node (SLN) metastasis in patients with breast cancer.

**Methods:**

A total of 164 breast cancer patients confirmed by pathology were prospectively enrolled from December 2017 to May 2018, and underwent DCE-MRI before surgery. Pharmacokinetic parameters and radiomics features were derived from DCE-MRI data. Least absolute shrinkage and selection operator (LASSO) regression method was used to select features, which were then utilized to construct three classification models, namely, the pharmacokinetic parameters model, the radiomics model, and the combined model. These models were built through the logistic regression method by using 10-fold cross validation strategy and were evaluated on the basis of the receiver operating characteristics (ROC) curve. An independent validation dataset was used to confirm the discriminatory power of the models.

**Results:**

Seven radiomics features were selected by LASSO logistic regression. The radiomics model, the pharmacokinetic parameters model, and the combined model yielded area under the curve (AUC) values of 0.81 (95% confidence interval [CI]: 0.72 to 0.89), 0.77 (95% CI: 0.68 to 0.86), and 0.80 (95% CI: 0.72 to 0.89), respectively, for the training cohort and 0.74 (95% CI: 0.59 to 0.89), 0.74 (95% CI: 0.59 to 0.90), and 0.76 (95% CI: 0.61 to 0.91), respectively, for the validation cohort. The combined model showed the best performance for the preoperative evaluation of SLN metastasis in breast cancer.

**Conclusions:**

The model incorporating radiomics features and pharmacokinetic parameters can be conveniently used for the individualized preoperative prediction of SLN metastasis in patients with breast cancer.

## Background

Breast cancer is a common malignancy in women and a major cause of cancer deaths [1]. Axillary lymph node status is one of the strongest prognostic factors in patients with breast cancer and is crucial for the treatment of this disease [2]. Since the 1990s, the sentinel lymph node biopsy (SLNB) for breast cancer has replaced axillary lymph node dissection (ALND) as the standard of care for primary treatment of early breast cancer [3]. However, this method is invasive and carries the risk of dye allergies and false negative results [4]. Therefore, a noninvasive and accurate method for the detection of sentinel lymph node (SLN) metastasis would be crucial in avoiding unnecessary postoperative complications and selecting the optimal therapy in clinical practice.

Breast cancer generally features distinct histological, molecular, and clinical phenotypes and may manifest as radiologic heterogeneity. Radiomics can transform image data into high-resolution image feature data that can be mined and provide deep quantitative information that cannot be determined by the naked eye of clinicians [5]. Radiomics is predictive of malignancy, response to neoadjuvant chemotherapy, prognostic factors, molecular subtypes, and recurrence risk in breast cancer [6, 7] and thus shows promising use in assessing and predicting SLN metastasis in tumors [8, 9].

Magnetic resonance imaging (MRI) has been widely used in the diagnosis and staging of breast cancer because of its advantages of non-radiation, high soft tissue contrast and functional imaging. Although studies have showed that MRI has superior performance than some other techniques such as ultrasound or mammography, its efficacy in identifying axillary lymph node status is unsatisfactory [10, 11]. Dynamic contrast enhanced magnetic resonance imaging (DCE-MRI) can provide pharmacokinetic parameters, including semiquantitative and quantitative parameters, and is a sensitive technique that reflects the extent of tumor angiogenesis [12]. Previous studies have demonstrated that pharmacokinetic parameters can potentially be used as prognostic or predictive biomarkers [13]. Bahri et al. [14] found that the Ktrans and Ke*p* values of metastatic lymph nodes are higher than those of nonmetastatic lymph nodes in breast cancer. However, using combined pharmacokinetic parameters and DCE-MRI radiomics features to predict SLN metastasis has not yet been demonstrated.

Therefore, the aim of our study was to construct and validate a noninvasive model from preoperative DCE-MRI to predict SLN metastasis in patients with breast cancer.

## Methods

### Patients

This prospective study was approved by the ethics committee, and written informed consent was obtained from all patients. We identified 257 consecutive patients with newly histologically proven invasive breast cancer from December 1, 2017 to May 1, 2018. The inclusion criteria were 1) had undergone pathological evaluation of SLN; 2) cancer focus with longest diameter > 5 mm; and 3) single mass enhancement. Exclusion criteria were 1) nonmass-like enhancement on DCE-MR images; 2) incomplete clinical or pathologic characteristics; and 3) undergone radiation therapy or chemotherapy treatment. In this study, all cases underwent axilla ultrasound. Patients with negative axilla by ultrasound underwent SLNB at the time of surgery. If the SLN was positive, ALND was performed. If patient was found to have suspicious positive axillary lymph node by ultrasound, an ultrasound-guided fine needle biopsy was performed. If there was a histologically positive lymph node on needle biopsy, the patient received ALND. If the biopsy result was negative, the patient received a SLNB.

Finally, 164 patients (78 positive for SLN and 86 negative for SLN) were included in this study. The training cohort included 124 cases from December 2017 to March 2018, and the validation cohort included 40 cases from April 2018 to May 2018.

### MRI acquisition

All images were obtained with a 3.0 T MRI system (GE Discovery 750 W) using an 8-channel breast-dedicated coil in prone position. The MRI sequences included axial T1-weighted imaging (T1WI) (repetition time [TR]/echo time [TE] = 520 ms/9 ms, slice thickness/gap = 5 mm/1 mm) and axial T2-weighted imaging (T2WI) (TR/TE = 5200 ms/90 ms, slice thickness/gap = 5 mm/1 mm). DCE-MRI was performed with volume acceleration sequence in the axial plane (TR/TE = 6.2 ms/2.3 ms; slice thickness/gap = 2 mm/0 mm; FOV = 360 × 360 mm^2^; matrix = 288 × 320; and flip angles: 5°, 10°, and 15°). The contrast agent (Omniscan, 0.2 mmol/kg body) was injected after the acquisition of one set of precontrast images by using a high-pressure syringe at a rate of 2.8 ml/s, followed by the injection of equivalent volume of saline at the same rate to wash out the residual contrast agent in the tube. The scanning time of each phase was 16 s for a total of 30 phases. In this study, we used the peak enhanced phase of the multiphase contrast-enhanced MRI selected in accordance with the time intensity curve because the image lesions had the largest amount of contrast with the background [15].

### Pharmacokinetic parameter extraction

DCE-MRI data were transferred to an off-line workstation and analyzed by using specialized quantitative analysis software (Omni Kinetics; GE Healthcare, China, Shanghai). Two experienced radiologists (with 10 years of experience in breast imaging diagnosis) who were blinded to the histopathological results independently performed the data analysis. Before parameter calculation, a nonlinear registration framework utilizing the Free Form Deformation algorithm was used to correct misalignment caused by body motion between consecutive DCE scans, and signal intensity was converted into omniscan concentration using the variable flip angle method. An arterial input function was extracted by manually drawing on the thoracic aorta. The two-compartment extended Tofts model was selected to calculate pharmacokinetic parameters, including the quantitative parameters volume transfer constant (Ktrans), reverse reflux rate constant (Kep), volume fraction of extravascular extracellular space (Ve), and volume fraction of plasma (Vp), as well as semiquantitative parameters, including time to peak (TTP), maximum concentration (MaxCon), maximal slope (MaxSlope), and area under curve (AUC). The entire tumor maximum layer was selected as the region of interest (ROI), and necrotic or cystic areas were excluded from the evaluation.

### Radiomics feature extraction

**Fig. 1:**
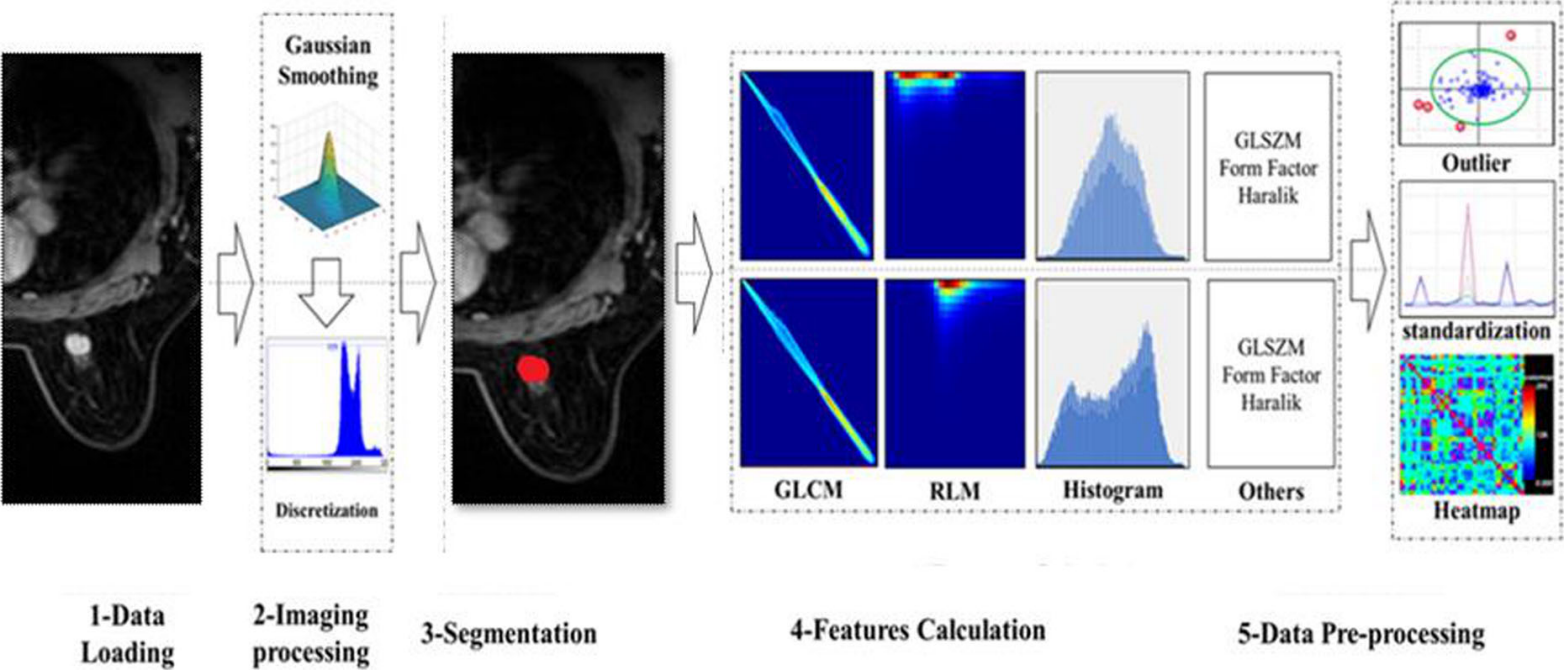
Radiomics workflow

Prior to image feature extraction, the original MRI image must be preprocessed; preprocessing steps include MRI signal intensity standardization and Gray-level quantization [16, 17]. On the basis of time signal intensity curves, the strongest enhanced phase was selected and the ROI was manually drawn by a radiologist with more than 10 years of experience and who was blinded to the pathological results (Fig. 1). As shown in Table 1, a total of 396 radiomics features classified as first-order, shape, and texture features were extracted from the ROI. This process was performed with AK software (Artificial Intelligence Kit; GE Healthcare, China, Shanghai).

**Table 1:** Radiomic features derived from the images

Calculation Methods	Radiomics Features	Feature Numbers
Histogram	Frequency size, Quantile, Variance, Kurtosis, Skewness, etc.	42
GLSZM	Size Zone Variability, Large Area Emphasis, High Intensity Emphasis etc.	11
Haralick matrix	HaraEntroy, Contrast, Inverse Difference Moment, Sum Average, Sum Variance	10
Form factor matrix	Maximum 3D Diameter, Spherical Disproportion, Sphericity, Surface Area, etc.	9
GLCM	ClusterProminence_AllDirection_offset1, Correlation_AllDirection_offset1, GLCMEnergy_angle45_offset4, etc.	144
RLM	Grey Level Non-Uniformity All Direction, High Grey Level Run Emphasis Angle Offset, Run Length Non-uniformity Angle Offset, etc.	180

### Feature selection

Intra- and inter-observer agreement was analyzed on the basis of intra- and inter-class correlation coefficient (ICCs) for all radiomics features extraction. A total of 30 random patients were selected, and radiologist A and radiologist B extracted the features of these 30 patients. Radiologist A then repeated the same procedure 1 week later. The ICCs greater than 0.75 indicated good consistency.

The LASSO logistic regression algorithm, which is suitable for the regression of high-dimensional data, was used to select the most useful predictive features from the primary data set.

### Predictive model building

The enrolled cases were randomly divided into two independent subsets at a ratio of 3:1, wherein 124 patients were used as the training cohort, and 40 patients were used as the independent validation cohort.

The logistics regression models were used to establish the radiomics model, the pharmacokinetic parameters model, and the combined model to predict SLN metastasis in breast cancer. The predictive efficiency of the predictive model was assessment by using receiver operating characteristics (ROC) curve in the training and validation cohorts.

### Statistical analysis

Patients age and tumor diameter were compared between the SLN-positive and SLN-negative groups by t test. Histological grade and molecular subtype were tested for trends using the chi-square test. All numerical data were presented as mean standard deviation. LASSO logistic regression was performed on the basis of 10-fold cross validation. Multivariate logistic regression was used to develop three models: the radiomics model, pharmacokinetic parameters model, and the combined model. ROC curve were used to evaluate the diagnostic performance of the three models. Sensitivity, specificity, and accuracy were calculated for each model. A two-sided *p* value of less than 0.05 was considered to indicate significant difference. All statistical tests were carried out in R3.5.1.

## Results

### Clinical characteristics

As shown in Table 2, age, tumor diameter, histological grade, and molecular subtype did not significantly differ between the SLN-positive and SLN-negative groups (*P* > 0.05).

**Table 2:** Clinical and Histopathological Characteristics

	Patients with positive SLN (*n* = 78)	Patients with negative SLN(*n* = 86)	*p* value
Age (mean ± SD)	55.71 ± 8.6	54.40 ± 11.1	0.483
Tumor size (mean ± SD)	2.24 ± 1.0	2.21 ± 1.2	0.698
Histological grade			0.171
I	4 (5.15%)	25 (11.6%)	
II	33 (42.3%)	41 (47.7%)	
III	41 (52.6%)	20 (40.7%)	
Molecular subtype			0.220
Luminal A	30 (38.5%)	31 (36%)	
Luminal B	37 (47.4%)	33 (38.4%)	
HER2 over-expression	6 (7.7%)	8 (9.3%)	
Basal-like	5 (6.4%)	14 (16.3%)	

### Feature selection and predictive performance of the model

The intra-observer ICC ranged from 0.869 to 0.894, and the inter-observer ICC ranged from 0.851 to 0.926, indicating favorable feature extraction reproducibility.

**Fig. 2:**
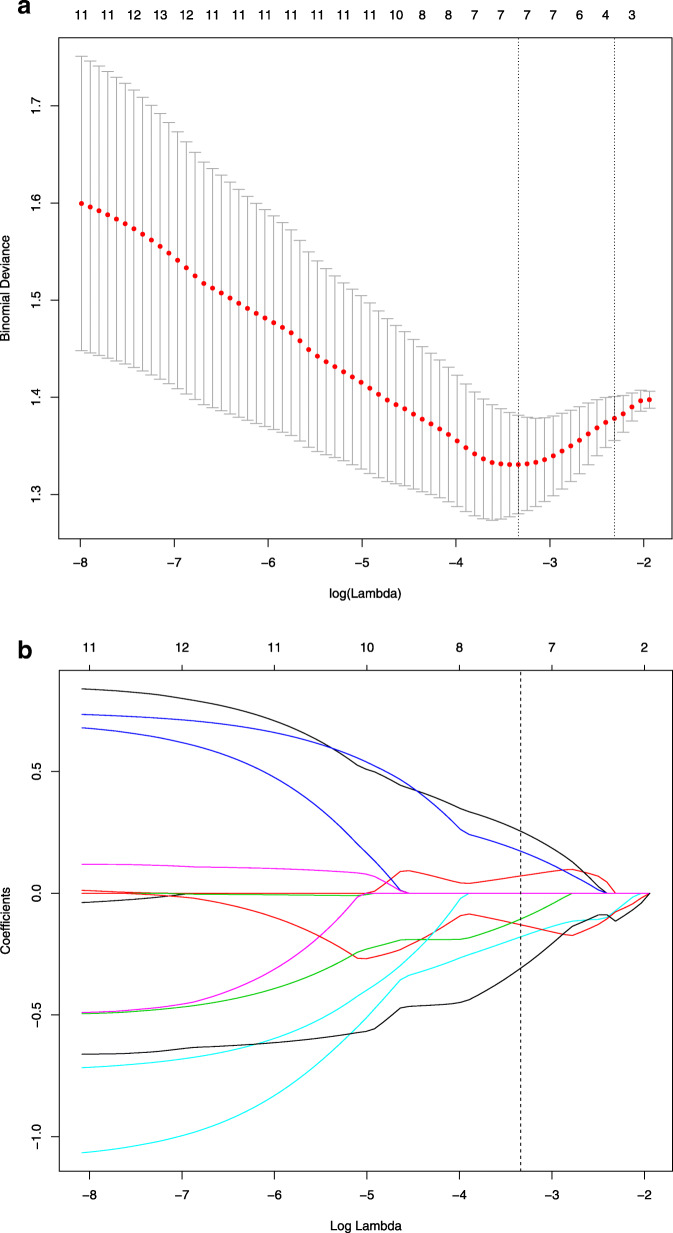
LASSO algorithm for feature selection. **a** Selection of adjustment parameters (lambda) in the LASSO model used 10-fold cross-validation via minimum criteria; **b** LASSO coefficient profiles of the features against the log (λ)

The training cohort had 396 selected radiomics features for LASSO logistic regression analysis. A total of seven radiomics features including Sum Entropy, Compactness-1, Inertia, Cluster Prominence, Correlation, GLCM Entropy, Kurtosis were selected by the LASSO logistic regression model (Fig. 2). SLN metastasis prediction models were developed using a multivariate logistic regression model based on pharmacokinetic parameters (Ktrans, Kep, Ve, Vp, TTP, MaxSlope, AUC, and MaxCon) and radiomics features.

**Fig. 3:**
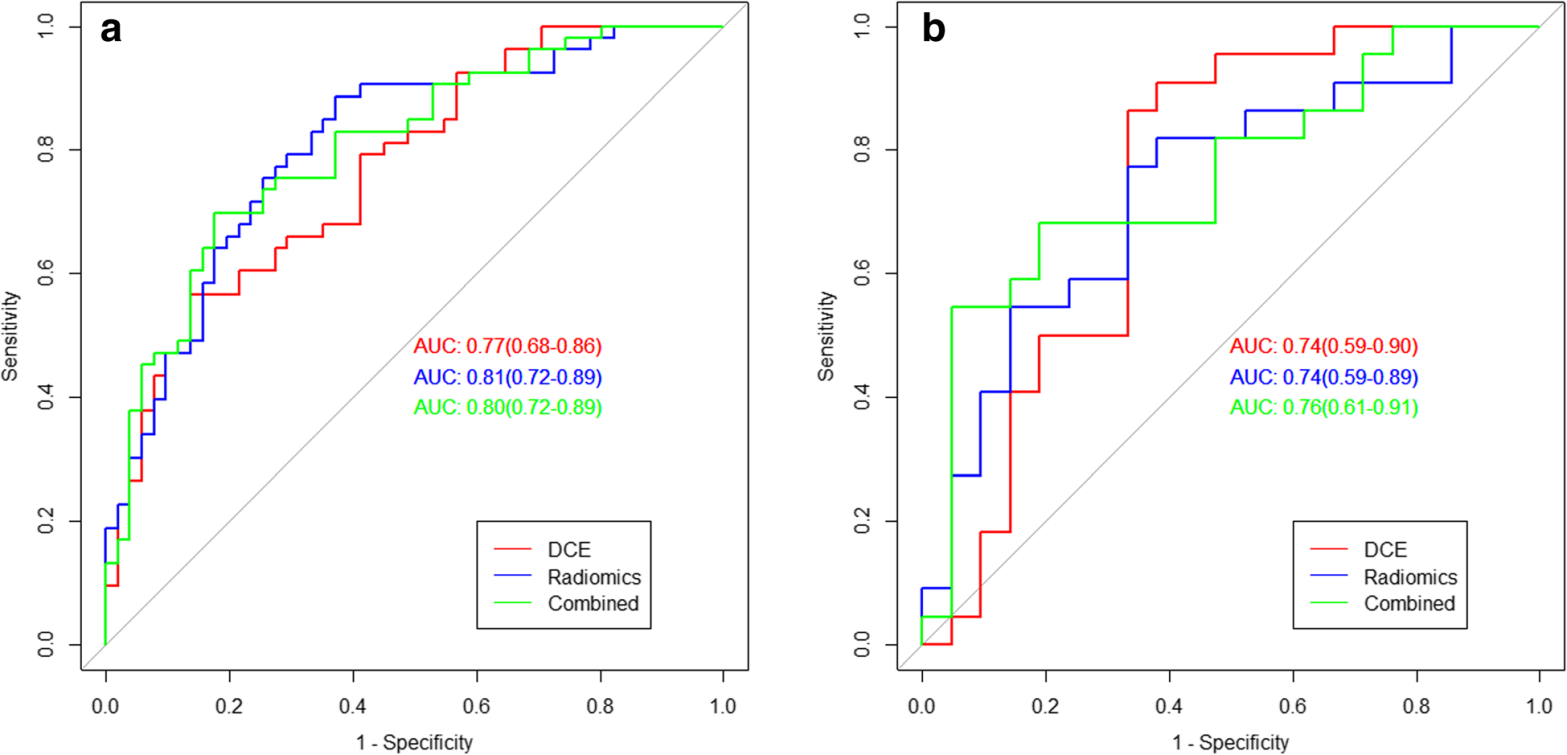
ROC curves of prediction models in the training (**a**) and validation (**b**) cohorts

In the training cohort, the radiomics model, pharmacokinetic parameters model, and the combined model yielded AUC values of 0.81 (95% CI: 0.72 to 0.89), 0.77 (95% CI: 0.68 to 0.86), and 0.80 (95% CI: 0.72 to 0.89), respectively. In the validation cohort, the pharmacokinetic parameters model, the radiomics model, and the combined model yielded AUC values of 0.74 (95% CI: 0.59 to 0.89), 0.74 (95% CI: 0.59 to 0.90), and 0.76 (95% CI: 0.61 to 0.91), respectively (Fig. 3).

The results for the validation cohort showed that the accuracy, sensitivity, and specificity of the pharmacokinetic parameters model were 69, 71, and 77%, respectively. The accuracy, sensitivity, and specificity of the radiomics model were 67, 64 and 79%, respectively. The accuracy, sensitivity, and specificity of the combined model were 76, 72 and 81%, respectively. The combined model results showed that the effect was higher than another (Table 3).

**Table 3:** Diagnostic performance of validation cohort

	accuracy	sensitivity	specificity
pharmacokinetic parameters model	0.69	0.71	0.77
Radiomics model	0.67	0.64	0.79
Combined model	0.76	0.72	0.81

## Discussion

In this study, the multivariate logistic regression model was used to establish prediction models for predicting SLN metastasis. The results showed that the combination of radiomics and hemodynamic characteristics can obtain an improved preoperative prediction model with promising performance in the prediction of SLN metastases with an AUC of 0.76 in the validation set. These results might help clinical decision-making with respect to axillary surgery, potentially avoiding invasive procedures in patients at a low risk of SLN metastases.

Assessing axillary lymph node metastasis as early as possible is essential for breast cancer surgical planning, adjuvant therapy planning, and prognostication. Determining axillary lymph node status remains a mandatory requirement of diagnostics. The SLNB and ALND are common methods for the estimation of axillary lymph node status. However, they are invasive [18]. Therefore, a noninvasive approach with high accuracy is necessary to preoperatively evaluate SLN metastasis. Developing a tool that accurately and noninvasively predicts axillary lymph node metastasis preoperatively provides great advantage. Yang used the radiomics of mammography to preoperatively predict SLN metastasis [19]. However, X-ray is limited by difficult detection of the complete picture of the lesion, the easy overlap between the lesion site and glandular tissue, and the easily missed diagnosis. MRI can completely show the type, range, and internal structure of lesions and clearly show the multicenter lesions. At the same time, DCE-MRI has a good diagnostic effect for tumor recurrence foci and multicentric cancer [20].

DCE-MRI can provide multiple pharmacokinetic parameters, including semiquantitative parameters and quantitative parameters, which can reveal the perfusion and vascular distribution of tissue at the molecular level [21]. The DCE-MRI-derived parameters Ktrans and Kep enable the estimation of tumor angiogenesis and proliferation in breast cancer [22]. Previous studies show that quantitative parameters can improve diagnostic accuracy and provide insight into the underlying biological characteristics of breast lesions [23]. Compared with semiquantitative parameters, quantitative parameters are less affected by the wide variability in MRI scanners, scanning sequence, temporal resolution, contrast media injection, and image postprocessing calculation [24]. In this study, we combined quantitative parameters (Ktrans, Kep, Ve, and Vp) with semiquantitative parameters (TTP, MaxSlope, AUC, and MaxCon), and demonstrated satisfactory performance in the diagnosis of SLN metastasis with an AUC of 0.77 in the training dataset and AUC of 0.74 in the validation dataset; these results indicate that the good effect of our model for the differentiation of SLN metastasis is better than that of previously reported methods [25].

Radiomics, an emerging technique that can convert digital medical images into mineable data for analysis for texture feature extraction, helps characterization within heterogeneous tumor lesions [26]. This approach can help clinicians improve detection, diagnosis, stage, and prediction power. Only a few studies have shown that DCE-MRI can predict the occurrence of SLN metastasis with high accuracy by radiomics [27, 28]. In 2018, Liu first attempted to predict SLN metastasis in breast cancer noninvasively by using DCE-MRI radiomics and demonstrated promising prediction performance in an independent validation set [29]. However, this study was retrospectively designed, whereas our study was a prospective study with radiomics from breast cancer. Our study sequence was standardized, and our evidence for clinical application was stringent. Previous studies only used DCE-MRI for feature extraction, and the accuracy of the comprehensive analysis of pharmacokinetic parameters was unknown. In our study, the model that combined radiomics features and pharmacokinetic parameters with an AUC of 0.76 in the validation cohort had higher prediction ability than any single model. The combined model indicates microcirculation function and information regarding tissue morphological characteristics, which can provide a comprehensive description of the tumor.

Recently, radiomics analysis based on other MRI sequences has been reported. In 2017, Dong applied T2-WI and DWI texture features to predict SLN metastasis [30]. Most of lesions on DWI and T2W images show reduced resolution and exacerbated distortion, and segmenting the lesions completely is difficult. In this study, DCE-MRI was used to derive DCE parameters and radiomics analysis, which clearly show the lesion boundaries. DCE-MRI has numerous scanning phases. Han et al. [28] extracted radiomics features from the axial first phase of the T1-weighted images of the DCE images of primary tumors to predict SLN metastasis. In this study, we used the peak enhanced phase of multiphase contrast enhanced MRI selected in accordance with the time intensity curve, which show the lesion boundaries clearly and have the greatest association with potential tissue information [15].

This study has several limitations. First, it is a preliminary exploratory study with a small sample size, and a large sample is necessary. Second, the manual method was applied to image segmentation although the satisfactory inter- and intra-observer reproducibility of radiomics features extraction was achieved. Moreover, over 90% of features had good reproducibility [31]. However, the automated method for image segmentation may have high stability [32, 33]. Third, our study lack external validation for the model. Multicenter validation is needed to acquire high-level evidence for clinical application. We will continue our research in future studies.

## Conclusions

In conclusion, our prospective research shows that pharmacokinetic parameters and the radiomics combined model represent a noninvasive predictive tool that shows good application prospects for the detection of SLN metastasis in patients with breast cancer. A multicenter validation study with a large sample size should be conducted to improve efficiency in subsequent works.

## Data Availability

The processed data required to reproduce these findings cannot be shared at this time as the data also forms part of an ongoing study.

## Abbreviations

DCE-MRI: Dynamic contrast enhanced magnetic resonance imaging
SLN: Sentinel lymph node
LASSO: Least absolute shrinkage and selection operator
Ktrans: Quantitative parameters volume transfer constant
Kep: Reverse reflux rate constant
Ve: Volume fraction of extravascular extracellular space
Vp: Volume fraction of plasma
TTP: Time to peak
MaxCon: Maximum concentration
MaxSlope: Maximal slope
ROC: Receiver operating characteristics
AUC: Area under curve
ROI: Region of interest
ICCs: Intra- and interclass correlation coefficient
CI: Confidence interval
